# Commentary on Thoral et al. (2024) ‘The relationship between mitochondrial respiration, resting metabolic rate and blood cell count in great tits’

**DOI:** 10.1242/bio.061770

**Published:** 2024-11-20

**Authors:** Kasja Malkoc, Michaela Hau, Scott McWilliams, Edyta Teresa Sadowska, Maciej Dzialo, Barbara Pierce, Lisa Trost, Ulf Bauchinger, Eve Udino, Stefania Casagrande

**Affiliations:** 1 Max Planck Institute for Biological Intelligence, Evolutionary Physiology Group, 82319 Seewiesen, Germany; 2 University of Konstanz, Konstanz, Germany; 3 University of Rhode Island, Department of Natural Resources Science, Kingston, RI 02881, USA; 4 Jagiellonian University, Institute of Environmental Sciences, 30-387 Kraków, Poland; 5 Sacred Heart University, Department of Biology, Fairfield, CT 06825, USA; 6 Max Planck Institute for Biological Intelligence, Department for Behavioral Neurobiology, 82319 Seewiesen, Germany; 7 Nencki Institute of Experimental Biology, PAS, 02-093 Warsaw, Poland

In a recent study, Thoral et al. (2024) explored the link between whole-organism resting metabolic rate (RMR) and mitochondrial respiration in red blood cells of wild great tits (*Parus major*). They captured great tits in winter to measure their night-time RMR with traditional respirometry, at thermoneutrality (20°C) for the first 4 h, and at 8°C for the remaining 4 h of measurements. Immediately after RMR measurements birds were blood-sampled and mitochondrial respiration states assessed in red blood cells at 41°C with high-resolution respirometry. RMR at 20°C and mitochondrial respiration (ROUTINE) did not show the expected positive relationship in intact cells, in which mitochondrial function relies on endogenous substrates. Yet, they identified a positive relationship between RMR at 20°C and phosphorylating respiration (OXPHOS CI+CII) in permeabilized cells, which are (non-viable) cells with modified plasma membranes that permit the entry of respiratory substrates, supplied at saturating amounts. However, RMR at 8°C and mitochondrial respiration in intact cells (ROUTINE) tended to be positively related (*P*=0.065, results provided in the supplemental materials) and it also again covaried positively with OXPHOS CI and OXPHOS CI+CII in permeabilized cells (*P*=0.037 and *P*=0.023, respectively). The authors concluded that a relationship between RMR and blood mitochondrial respiration traits exists, but that it remains hidden when measuring intact cells and only emerges in permeabilized cells. These results are in contrast to two recent studies, also on great tits (Malkoc et al., 2021) and on European starlings (*Sturnus vulgaris*; Casagrande et al., 2023), which found positive associations between RMR and mitochondrial respiration in intact cells. We fully agree with the call by Thoral et al. for more validations of the use of mitochondrial respiration as a proxy for whole-organism metabolic rate. This commentary is meant to highlight potential reasons for the divergent findings in the three studies, particularly on the correlation between RMR and mitochondrial respiration in intact blood cells, and to develop best practices for the use of this exciting and promising technique.

i) *Using intact versus permeabilized cells*. Measuring mitochondrial metabolism in living, intact cells, which rely on their natural reserves and maintain processes of membrane transport as in their tissue of origin, holds a considerable potential for ecological studies. For example, in Thoral et al. ROUTINE and RMR tended to be positively correlated when RMR was measured at 8°C, and not at 20°C. Indeed, not only does a temperature of 8°C better reflect the natural ecological conditions experienced by the wintering great tits used in the study, but also, RMR at this temperature was measured in closer proximity to blood sampling, which ensured that individual's physiological status was more similar between the two measures. Conversely, permeabilization and substrate saturation removes effects of resource limitation and membrane transport dynamics, representing a more artificial situation (which has its merits, see below). Cell permeabilization represents a state never experienced by cells *in vivo*, unless their cell membrane is damaged. Such artificial conditions may lead ecologists to question the ecological relevance of mitochondrial measures in permeabilized cells. For example, the use of substrates should be validated to test whether malate and pyruvate alone are used by erythrocytes' complex I in wintering great tits, and not other substrates like glutamate or even β-oxidation products like palmitoyl or carnitine, as recently shown in muscles of migratory birds (Rhodes et al., 2024). Exploring the relationship between ROUTINE and OXPHOS could prove crucial to establish how much mitochondrial function is affected by the cell's condition. For instance, Thoral et al. (2024) could have investigated the correlation between their measures of ROUTINE (quantified when cells were still intact) and of OXPHOS (quantified after permeabilizing the cells).

ii) *Effects of temperature on metabolic rates*. While all three studies used similar *in vitro* temperatures for measuring mitochondrial metabolic rate (40-41°C), the temperatures to which the birds were acclimatized prior to the experiment and those they experienced during RMR measurements differed substantially (Casagrande et al., 2023; Malkoc et al., 2021; Thoral et al., 2024)^1^, resulting in different temperature dynamics before and after the blood sampling. Similarly to RMR, which increases with greater thermoregulatory costs (Swanson and Olmstead, 1999), mitochondrial respiration is strongly affected by changes in environmental temperature (Seebacher, 2009). However, it seems likely that the divergent temperature regimes in the three studies have affected organismal and cellular respiration rates differently, as well as their relationship. Therefore, in future validation studies, considerable research effort should be directed to investigate how environmental temperature, acclimatization state, body and assay temperatures affect RMR and mitochondrial metabolic rate.

iii) *Effects of stressors on metabolic rates*. Malkoc et al. (2021) showed that high, stress-induced corticosterone concentrations resulting from the experimental procedure (i.e. catching, handling and confinement in metabolic chambers), can reverse the positive relationship between metabolic rate measured at the organismal and cell levels. This could be attributed to the regulatory role of elevated corticosterone by which non-immediately essential tissues, including blood, are prevented access to blood substrates. Indeed, in this case, ‘feeding’ blood cells with exogenous substrates (as done in permeabilization protocols) could be a valuable way to test for the effects of tissue-specific resource availability. However, care should be taken when using mitochondrial substrates, as species can differ in their utilization, necessitating proper validation to ensure ecologically meaningful results (Metcalfe et al., 2023). Measuring plasma glucocorticoid concentrations from the same blood sample from which mitochondrial metabolism is quantified (within the species-specific time after which glucocorticoids begin to rise, for example within the 3 min following the removal from the metabolic chamber; Romero and Reed, 2005) is especially important when mitochondrial respiration is measured in laboratory conditions where study individuals are typically exposed to a series of stressful events. This measurement would have been useful in Thoral et al. to account for (a) differences in stress status among individuals at the time of sampling^2^, (b) its (potentially divergent) effects on RMR and ROUTINE and (c) to facilitate comparison with other studies^3^ to increase our understanding of mitochondrial bioenergetics and its links with stress.

We appreciate Thoral et al. (2024) for advancing our understanding of blood mitochondrial bioenergetics, and emphasize that their study marks a step forward in the field. Indeed, their documented relationship between the oxygen consumption by permeabilized cells and the organism as a whole, pinpoints substrate saturation as an experimental procedure that may allow us to address mechanistic questions that do not require a cell to be in its natural state or to overcome context-dependent nutrient limitation. However, for increasing our understanding of the metabolic challenges that animals face in the wild, knowing how intact cells regulate their energy metabolism may be most relevant.

## Footnotes

1. Acclimatized to winter temperatures versus RMR being measured at 20 and 8°C in Thoral et al.; acclimatized to winter temperatures that were maintained during RMR measurements in Malkoc et al.; and acclimatized to autumn temperatures versus RMR being measured at 25°C in Casagrande et al.

2. In Thoral et al, up to 3 h could elapse daily between catching and the start of respirometry. Consequently, birds caught at the beginning of the catching session likely experienced stressful conditions for a much longer duration than those caught at the end.

3. For example, the cumulative stress experienced by the birds at the time of blood sampling differed among the three studies due to a different duration of pre-respirometry experimental procedures. While in Malkoc et al. and Casagrande et al., respectively, 40 and 20 minutes passed between catching and the start of respirometry, in Thoral et al. these procedures could take up to 3 h.
